# Drug repurposing for the treatment of glioblastoma multiforme

**DOI:** 10.1186/s13046-017-0642-x

**Published:** 2017-11-28

**Authors:** Claudia Abbruzzese, Silvia Matteoni, Michele Signore, Luca Cardone, Kavindra Nath, Jerry D. Glickson, Marco G. Paggi

**Affiliations:** 1 Department of Research, Advanced Diagnostics and Technological Innovation, Unit of Cellular Networks and Therapeutic Targets, Proteomics Area, Regina Elena National Cancer Institute, IRCCS, Via Elio Chianesi, 53, Rome, Italy; 2 RPPA Unit, Proteomics Area, Core Facilities, Istituto Superiore di Sanità, Rome, Italy; 3 Department of Research, Advanced Diagnostics and Technological Innovation, Unit of Cellular Networks and Therapeutic Targets, Regina Elena National Cancer Institute, IRCCS, Rome, Italy; 4 Laboratory of Molecular Imaging, Department of Radiology, University of Pennsylvania Perelman School of Medicine, Philadelphia, PA USA

**Keywords:** Drug repurposing, Drug repositioning, Glioblastoma multiforme, Cancer treatment, High-throughput technologies, Signal transduction, Energy metabolism

## Abstract

**Background:**

Glioblastoma Multiforme is the deadliest type of brain tumor and is characterized by very poor prognosis with a limited overall survival. Current optimal therapeutic approach has essentially remained unchanged for more than a decade, consisting in maximal surgical resection followed by radiotherapy plus temozolomide.

**Main body:**

Such a dismal patient outcome represents a compelling need for innovative and effective therapeutic approaches. Given the development of new drugs is a process presently characterized by an immense increase in costs and development time, drug repositioning, finding new uses for existing approved drugs or drug repurposing, re-use of old drugs when novel molecular findings make them attractive again, are gaining significance in clinical pharmacology, since it allows faster and less expensive delivery of potentially useful drugs from the bench to the bedside. This is quite evident in glioblastoma, where a number of old drugs is now considered for clinical use, often in association with the first-line therapeutic intervention. Interestingly, most of these medications are, or have been, widely employed for decades in non-neoplastic pathologies without relevant side effects. Now, the refinement of their molecular mechanism(s) of action through up-to-date technologies is paving the way for their use in the therapeutic approach of glioblastoma as well as other cancer types.

**Short conclusion:**

The spiraling costs of new antineoplastic drugs and the long time required for them to reach the market demands a profoundly different approach to keep lifesaving therapies affordable for cancer patients. In this context, repurposing can represent a relatively inexpensive, safe and fast approach to glioblastoma treatment. To this end, pros and cons must be accurately considered.

## Background

Glioblastoma multiforme (GBM) is the most frequent and lethal brain tumor. Location, aggressiveness and diffuse infiltrative growth make GBM therapy extremely challenging and frequently unsuccessful. State-of-art first-line treatment of newly diagnosed cases, managed according to Stupp et al., namely maximal surgical resection followed by radiotherapy (RT) plus temozolomide (TMZ) [1], provides scarce benefits overall in clinical outcome. Indeed, median overall survival of GBM patients remains between 12 and 15 months, with a 5-year survival rate from diagnosis of less than 5% [2, 3]. Such a poor prognosis generates a compelling need for innovative and effective therapeutic strategies, ranging from hi-tech robotic surgery to the use of cutting edge radio- and chemotherapy.

Several novel chemotherapeutic strategies against GBM are thus frequently proposed, but current regulations for drug registration are producing an immense increase in cost and development time. At present, the pipeline from the identification of a potential molecular target up to drug registration and marketing [i.e.: discovery and screening/design, lead optimization (drug development), absorption, distribution, metabolism, excretion and toxicity (ADMET), clinical development (Phase I and Phase II trials), registration and marketing] ranges from 10 to 17 years [4], and the overall cost averages between 1 and 2 billion US$ [5]. Therefore, it is not surprising that drug repositioning, finding new uses for existing drugs, or drug repurposing, a re-use of old drugs when novel molecular findings make them attractive again, are gaining importance in clinical pharmacology. Currently, repositioning and repurposing are often used as synonyms. Indeed, mining within an enormous amount of already synthesized, and often already clinically employed, compounds can realistically cut research expenses and the overall development time to bring an effective drug to the clinics. Repurposing is also considered a safer approach, due to the often available pre-existing knowledge on dosage, safety and side effects of a drug, which would dramatically improve the effectiveness of a clinical trial. Thus, a rough estimate of costs and development time for repositioned drugs to reach the market is 300 million US$ and 6 years, respectively [5], which still appears not to be satisfactory, but definitely competitive in comparison to the development ab initio of new therapeutic agents.

Paradigmatic, well-known stories of success in drug repositioning include minoxidil [6], sildenafil [7, 8], thalidomide [9], azidothymidine [10] and many other compounds. For most repurposed drugs, the new therapeutic indications were understood and achieved serendipitously, although the recent -omics era is opening a door new exciting opportunities for molecular exploration of new, potential drug uses. For example, relevant molecular targets already targeted by an approved drug in the context of a specific pathology, may provide a reasonable starting point for repositioning this compound to novel therapeutic indications. This will speed up the use of potentially effective compounds and will increase the number of drugs which can be approved for use in infrequent diseases, as in rare cancer subtypes. Thus, increasing relevance is now being attributed to molecular signatures rather than to established classifications of the disease according to canonical pathological parameters.

Combining molecular signatures with drugs known to be effective in interfering with specific biochemical pathways is the ultimate challenge of molecular pharmacology, especially in the field of cancer therapy.

## Main text

GBM overall poor prognosis has been the major impetus for advancing extensive molecular characterization of this disease, with the aim of addressing specific treatments according to molecular subtypes [11–13]. Benefits from the Stupp regimen, for example, are most prominent in patients with O-6-methylguanine-DNA methyltransferase (MGMT) high methylation status, and, in addition, selective therapies can be used according to the IDH1/2 mutational status [14, 15]. Nevertheless, despite that the latest technological advancements have allowed considerable advances in molecular characterization of this form of cancer and the consequent use of novel experimental therapies, GBM prognosis remains dismal.

### GBM and targeted therapies

Recent proteogenomic studies have led to the identification of diverse molecular signatures and actionable signaling pathways in GBM, such as those targeting EGFR and PI3K [3, 16]. This is in line with the notion that EGFR and other described genomic alterations, as those involving BRAF [17] or the FGFR family [18], represent valuable drug targets, but the concomitant presence of different mutations/alterations within the same tumor may generate dissimilar, non-homogeneous responses toward a specific inhibitor. Indeed, the notorious GBM heterogeneity [19, 20] clearly represents a condition predisposing to drug resistance and disease relapse, since these clinical events are usually driven by cell types which either have been able to escape previous therapeutic approach(es) or were generated by clonal selection of mutations as a result of a Darwinian process [21].

Targeted therapies hold an unprecedented potential, but also conceal inherent limits. The use of such innovative strategies is often associated with considerably high costs for predicting therapeutic efficacy as well as those of therapeutic procedures. Moreover, targeted drugs often lead to dramatic, although short-lived, clinical benefits only in a restricted fraction of cancer patients [22]. Such a limited favorable outcome could possibly be faced using therapeutic strategies perhaps less specific, but designed to hit general cancer cell survival pathways, such as energy metabolism, inhibition of apoptosis, autophagy and inhibition of host immune response.

### Approaches for drug repositioning

Needless to say, in order to efficiently repurpose drugs that are already approved for human use, a careful selection is required, followed by thorough demonstration of their effectiveness in other biological contexts. We will now discuss some methods useful for selection of effective testing and repurposing of drugs in cancer therapy.

#### *Computational (*in silico*) drug repositioning*

This approach usually utilizes and elaborates public databases. Data come from basic and translational research, clinical trials, anecdotal reports regarding off-label uses and other published human data information available. Using artificial intelligence algorithms, as well as other bioinformatics tools, investigators systematically try to identify interaction networks between drugs and protein targets. Clearly in silico drug repositioning is a powerful technology and carries evident significant advantages, including speed and reduced costs [23, 24].

Computational approaches require the initial generation of a drug network based on drug-drug association. Several parameters are usually employed, such as similarity in molecular structure, active catalytic sites or ligand binding sites [25]. A promising approach is the generation of drug networks based on the similarity between compounds in perturbing gene expression signatures [26, 27]. This methodology presents three main advantages: i) robustness of algorithms for gene expression data analysis; ii) cost-effectiveness; iii) availability of freely accessible resources. In addition, the transcriptional profile following drug treatment is likely to integrate multiple levels of drug response, also those elicited by secondary targets of the compound tested. A drug network allows identifying i) drugs with similar mechanism of action (MoA), the first step for a potential repurposing; ii) MoA of orphan/new drugs on the basis of shared molecular signatures; iii) drugs eliciting a signature displaying reverse features, when compared with those associated with a disease and thus potentially able to rescue it [26].

Freely available resources are ArrayExpress [28], NCBI-GEO [29] and cMap [30], all large public repositories of gene expression data. Moreover, tools such as DAVID [31], MSigDB [32] or GeneSigDB [33] allow characterizing large gene lists by using pre-defined functional terms. This strategy has been found effective also for drug repositioning [34, 35].

Recently, drug repositioning has been able to complement and assist targeted therapeutics discovery. Indeed, bio-computational approach could be successfully implemented for repurposing therapeutics able to inhibit oncogenically activated molecular pathways that are known to have a well-established impact on the molecular pathogenesis of cancer. This approach is based on modeling specific molecular alterations in cell lines, followed by the generation of an oncogene-specific gene signature. This allows the inspection of drug network-associated signatures to reposition drugs able to “revert” the oncogenic signature, highlighting the possible role for these compounds as pathway inhibitors [27], as it has been successfully demonstrated for the identification of inhibitors of the PI3K/mTOR pathway [35, 36]. Since oncogenic pathway signatures delineate the oncogenic phenotype and represent the way to address targeted therapies [37, 38], the approach aiming at reverting oncogenic signatures could be exploited also for drug repositioning in GBM [39], also considering the profound knowledge achieved in the genomic landscape in this disease [3].

Nonetheless, before conceiving any clinical use, in vitro and mainly in vivo studies are mandatorily required in order to confirm computational predictions and design a clinical trial.

#### Activity-based drug repositioning

Activity-based drug repositioning essentially relies on protein target-based screenings, also in the absence of concurrent structural information. In the field of cancer therapy, molecular characterization has already allowed off-target use of specific inhibitors in pathologies different from those for which the agent was originally engineered and registered. Indeed, a paradigmatic shortcut is the discovery of actionable genomic events in a tumor type or in its subset and the straightforward possible use of a specific inhibitor already shown to be effective in other tumors carrying the same actionable mutation. For example, ALK inhibitors crizotinib or ceritinib and other second-generation inhibitors are dramatically effective in all tumors displaying dependence on an over-activated ALK kinase, usually due to the EML4-ALK rearrangement, such as anaplastic large cell lymphoma, neuroblastoma and non-small-cell lung carcinoma [40]. This represents a paradigmatic case scenario in which discoveries on the molecular basis of a disease provide an unprecedented and quick opportunity to translate research findings into new therapeutic approaches and medicines.

#### How to expand our knowledge of a drug

How much do we really know about the activity of a drug? Besides serendipity and apart from empirical or epidemiological observations, delving into the activity of a drug at a molecular level is now feasible. With the help of novel technologies in the field of sequencing, imaging, spectroscopy and spectrometry, basic sciences can investigate the MoA of a drug in depth and establish its potential clinical uses. The presence of quasi-unlimited experimental and computational opportunities (e.g. high-throughput platforms and high-performance computers) is timely pushing us towards the resolution of most issues related to drug repurposing. For example, only recently we were able to elucidate the real MoA of old drugs or even that of molecules belonging to the ancient pharmacopoeias (see below).

##### Genomics

The great expectations generated by the “genomic era” have been in part fulfilled. The production and elaboration, by Next Generation Sequencing (NGS) technologies, of whole exome, transcriptomic and epigenomic data, as well as their integration, is enabling us with rational personalized treatments, even using repositioned drugs [41], in patients affected by several types of cancer [42, 43], including GBM [44].

##### Proteomics

This approach is particularly relevant, since proteomics closely deals with the ground-level mechanisms underlying diseases and may provide appropriate therapeutic suggestions. Moreover, targeted proteomic profiles often demonstrate the lack of a direct relationship between static genomic data and the dynamic alterations of downstream signal transduction networks [3]. In this regard, Activity-Based Protein Profiling (ABPP), Reverse-Phase Protein microArrays (RPPA) and Magnetic Resonance Spectroscopy (MRS) are technologies that play a pivotal role in molecular pharmacology.

ABPP is capable of determining the function of several enzymes in complex biological systems using site-directed covalent probes aimed at assessing the functional state of specific classes of enzymes [45]. ABPP is gaining importance as a uniquely powerful post-genomic method for monitoring protein function as well as the molecular effects of drugs. ABPP proposes a variety of molecular probes able to monitor several enzyme activities, e.g. kinase, protease, serine hydrolase, metalloproteinase, cysteine protease, caspase, deubiquitylase, as well as Cytochrome P450 [46]. The use of this technology is often connected with Mass Spectrometry (MS) analysis. In clinical pharmacology, ABPP is particularly useful for understanding drug-target interactions, identifying new therapeutically relevant targets as well as off-target effects and, in addition, providing important information to assess a precise drug concentration required to hit (or not) secondary drug targets [41].

RPPA is a powerful, antibody-based technique that allows simultaneous profiling of diverse key regulatory cellular factors. RPPA was originally developed to investigate cancer cell signaling and is designed for relative and multiplexed quantification of specific cellular proteins along with their post-translational modifications [47, 48]. Opposed to MS, in RPPA complex protein matrices are immobilized onto a nitrocellulose substrate. Subsequent immunodetection, using total content as well as modification-specific antibodies, allows fine measurement of the functional state of selected cell signaling regulators. RPPA represents the state-of-art in supervised analysis of post-translational protein modifications and, in the context of repurposing, is an efficient tool to delineate the effects of drugs on several, cancer-related cellular pathways.

Essentially, while ABPP identifies the physical target(s) of a drug, RPPA evaluates the biological consequences of the drug activity. Indeed, the MoA of a drug cannot be predicted solely based on the detailed spectrum of known drug targets.

MRS is a non-invasive methodology for the analysis of flux through key pathways of cancer cell intermediary metabolism. MRS can measure flux of metabolites in glycolysis, Krebs cycle, pentose phosphate shunt, and anaplerotic pathways as pyruvate–malate shuttling, glutaminolysis, and fatty acid biosynthesis and oxidation [49]. In preclinical models, flux measurements, in the absence or presence of an interfering drug, can efficiently monitor the effect of a compound on a specific metabolic pathway in vivo. Given that this technology is non-invasive, the measurements of flux analysis can be obtained in the same subject, before and after drug administration, thus generating particularly reliable results.

In such a complex scenario, we should not ignore the possible (and frequent) repositioning of non-cancer-related drugs in antineoplastic therapy, as assessed by various empirical and/or experimental data. Some of the following examples clearly support this assertion.

### Disulfiram

Disulfiram is an ALDH1 inhibitor. Since it is able to block the production of acetic acid from ethanol, it is a medication that has been employed for decades in complete safety to treat alcohol abuse. ALDH1 is a staminal marker for GBM [50] and has been implicated in GBM resistance toward TMZ [51]. Acetate is an essential metabolite for the Krebs cycle and plays a key role in GBM and brain metastases bioenergetics [52]. It is an activator, through AMPK, of cellular energy metabolism, therefore, treatment of sensitive cells with disulfiram results in energy depletion. Disulfiram has increased activity if administered in concomitance to divalent cations (e.g. Cu gluconate). Of note, the effect of disulfiram appears also selectively oriented toward the stem cell compartment of a tumor [53, 54].

Interestingly, disulfiram has been recently described as an inhibitor of NF-κB [55] and MGMT [56, 57]. There are open clinical trials for the use of disulfiram (plus Cu gluconate), in association with other drugs, in GBM therapy (Table 1).

**Table 1:** NIH-approved clinical trials involving repurposed drugs (Metformin, Disulfiram, Chloroquine) for the treatment of GBM and other brain cancers

NCT Number	Title of the study	Disease	Interventions	Clinical Phase
NCT02780024	Metformin, Neo-adjuvant Temozolomide and Hypo- Accelerated Radiotherapy Followed by Adjuvant TMZ in Patients With GBM	GBM	•Drug: Metformin	Phase 2
NCT03151772	Bioavailability of Disulfiram and Metformin in Glioblastomas	GBM	•Drug: Disulfiram •Drug: Metformin	Early Phase 1
NCT01430351	Phase I Factorial Trial of Temozolomide, Memantine, Mefloquine, and Metformin for Post-Radiation Therapy (RT) Glioblastoma Multiforme (GBM)	Brain Cancer	•Drug: Temozolomide •Drug: Memantine •Drug: Mefloquine •Drug: Metformin	Phase 1
NCT02149459	Treatment of Recurrent Brain Tumors: Metabolic Manipulation Combined With Radiotherapy	Brain Neoplasms	•Radiation: Partial brain reirradiation. •Drug: Metformin •Behavioral: low carbohydrate diet	Phase 1
NCT01777919	Disulfiram/Copper Combination In The Treatment of Newly Diagnosed Glioblastoma Multiforme	GBM	•Drug: Temozolomide •Drug: Disulfiram •Drug: Copper	Phase 2
NCT01907165	Disulfiram in Treating Patients With Glioblastoma Multiforme After Radiation Therapy With Temozolomide	GBM	•Drug: Temozolomide •Drug: Disulfiram •Dietary Supplement: Copper gluconate	Early Phase 1
NCT02715609	Disulfiram/Copper With Concurrent Radiation Therapy and Temozolomide in Patients With Newly Diagnosed Glioblastoma	GBM	•Drug: Disulfiram •Drug: Copper Gluconate •Procedure: Surgery •Radiation: Radiation •Drug: Temozolomide	Phase 1 Phase 2
NCT03151772	Bioavailability of Disulfiram and Metformin in Glioblastomas	GBM	•Drug: Disulfiram •Drug: Metformin	Early Phase 1
NCT03034135	Safety, Tolerability and Efficacy of Disulfiram and Copper Gluconate in Recurrent Glioblastoma	Recurrent GBM	•Drug: Disulfiram/Copper	Phase 2
NCT02678975	Disulfiram in Recurrent Glioblastoma	Glioma and GBM	•Drug: Disulfiram •Dietary Supplement: Copper •Drug: Alkylating agents	Phase 2 Phase 3
NCT02770378	A Proof-of-concept Clinical Trial Assessing the Safety of the Coordinated Undermining of Survival Paths by 9 Repurposed Drugs Combined With Metronomic Temozolomide (CUSP9v3 Treatment Protocol) for Recurrent Glioblastoma	GBM	•Drug: Temozolomide •Drug: Aprepitant •Drug: Minocycline •Drug: Disulfiram •Drug: Celecoxib •Drug: Sertraline •Drug: Captopril •Drug: Itraconazole •Drug: Ritonavir •Drug: Auranofin	Phase 1
NCT00224978	Chloroquine for Treatment of Glioblastoma Multiforme	GBM	•Drug: Chloroquine	Phase 3
NCT02378532	The Addition of Chloroquine to Chemoradiation for Glioblastoma	GBM	•Drug: Chloroquine •Radiation: Radiotherapy •Drug: Temozolomide	Phase 1
NCT02432417	The Addition of Chloroquine to Chemoradiation for Glioblastoma	High-grade astrocytoma GBM	•Drug: Chloroquine	Phase 2

Source: https://clinicaltrials.gov/

### Rapamycin and derivatives

Rapamycin (Sirolimus), was isolated in the early ‘70s. It was first registered as an antifungal drug and was then employed as immunosuppressant after organ transplantation and also as anticancer agent. Indeed, the unifying MoA which possibly explains rapamycin’s multifaceted pharmacological spectrum of activity has been identified only recently as the inhibition of mTOR and, specifically, of the mTORC1 complex. These findings are summarized by Seto et al. [58]. In addition, a peculiar effect of rapamycin appears evident in in a mouse model of glioma-initiating cells [59].

Rapamycin also cures autoimmune lymphoproliferative syndrome (ALPS), a pre-cancerous status for which no therapy was previously available. Bride et al. report results of the first prospective multi-institutional trial of a long-term single-agent therapy for refractory cytopenias using rapamycin in 30 patients and showing remarkable efficacy in children with ALPS [60].

Several rapamycin derivatives (rapalogues) have been synthesized, e.g. temsirolimus, everolimus and ridaforolimus. Recently, rapamycin and its derivatives are gaining considerable interest due to their involvement in processes decelerating geroconversion, i.e. cellular ageing, [61, 62] and are also employed in Phase 2 clinical trials involving GBM patients showing therapeutic benefits in subjects whose tumor displays high mTORC1 activity [63].At present (November 2017), the site https://clinicaltrials.gov lists >30 clinical trials involving rapamycin or its derivatives in GBM and, more generically, in brain and CNS cancer therapy. Due to their number, these clinical trials will not be listed in Table 1.

### Metformin

Metformin is the most widely used oral antidiabetic drug and belongs, along with phenformin and buformin, to the group of biguanides. This drug has low and infrequent side effects (lactic acidosis in predisposed subjects) and, as a drug safety criterion, is now considered for its potential use in cancer chemoprevention [64–66]. Metformin activates AMPK and inhibits mTORC1 activity [67, 68], mainly due to its role in impeding the mitochondrial respiratory chain, thus increasing cytosolic AMP concentration. In this way, metformin reduces energy efficiency, thus increasing glucose consumption, which is, together with the inhibition of hepatic gluconeogenesis, the key mechanism responsible for its ability to lower glycaemia in diabetic patients. Recently, a set of kinases have been identified as potential metformin targets, including SGK1 and EGFR, but not AKT1 [69].

Metformin reduces aging in *C. elegans*, increasing its lifespan [64], while combined administration of metformin and rapamycin has been shown to be effective in increasing lifespan in mice [70].

The use of metformin at higher than conventional doses for antidiabetic therapy is postulated as a tool for reprogramming cancer cell metabolism, possibly providing new perspectives for synthetic lethality through intelligent drug combinations. Along similar lines, metformin could be used to target cells characterized by hypersensitivity to energetic stress [65], and is now finding its own role as an adjuvant in cancer therapy [71]. Currently, clinical trials are active for its use, in association with other drugs, in GBM therapy (Table 1).

### Lonidamine

In the late 1980s, lonidamine (LND, also known as AF1890), a reversible inhibitor of spermatogenesis [72], was widely employed in clinical trials for cancer therapy in combination with several anticancer drugs and/or radiation (for a review, see [73]). LND is characterized by relatively mild side effects that do not overlap with those related to conventional cytostatic therapies [74]. Its toxicity does not involve bone marrow, but myalgia appears as the most relevant and common side effect [74], likely related to the proton-linked Monocarboxylate Transporter (MCT) inhibition and consequent lactate accumulation in myocytes. In male patients, reversible azoospermia should be taken into account.

During its clinical use as an anticancer drug in combination with other therapeutic regimens, LND exhibited encouraging results in some contexts, such as Head & Neck cancer [74] and brain tumors [75, 76]. The drug was defined “unconventional” because the target of LND was at the level of energy metabolism, selectively producing drastic energy depletion in cancer cells in vitro and in vivo [73].

The rationale underlying the repurposing of LND originates from a more accurate and intriguing identification of its MoA. Now, further evidence, using the most recent technologies, mainly MRS, refines the MoA of the drug, showing that LND concurrently inhibits L-lactic acid efflux from cells mediated by the MCT family proteins. In addition, recent data on Mitochondrial Pyruvate Carrier (MPC) and complex II inhibition [77, 78] strongly support previous reports on the decrease of oxygen consumption by the drug [79, 80]. LND has also been demonstrated to elicit a cytotoxic autophagic response in GBM cells [81].

Used as a single agent, LND does not efficiently impede cancer cell growth, both in vitro and in vivo, being its effects transient and reversible. Nevertheless, when administered in association with other anticancer radio- or chemotherapeutic schemes, LND interferes with the survival pathways from which cancer cells are dependent. This is essentially due to the potent ATP depletion and acidification of the intracellular environment produced by LND, both phenomena being far more intense in cancer than in normal tissues [82].

Interestingly, similar to metformin, LND has been demonstrated to reduce *C. elegans* aging [83].

### Chloroquine and related antimalarial drugs

This class of drugs has been widely used for decades in malaria prevention and therapy. These molecules are effective in blocking *P. falciparum* life cycle and are relatively well-tolerated. Recently, antimalarial drugs are also considered in cancer chemotherapy [84]. Indeed, a key role has been attributed to these drugs in inhibiting the late steps of autophagy. Autophagy is a homeostatic intracellular process which enables the degradation of old or damaged intracellular organelles. In normal cells, autophagy acts as a type of rejuvenation procedure, while in cancer cells, and mainly cancer stem cells, it provides a noticeable and self-generated source of energy [85]. When the autophagic process is evoked, the final cellular outcome can be quite diverse, ranging from a cytotoxic effect, eventually culminating in cell death, to an increase in survival capabilities in an unfavorable environment. In tumor cells, autophagy is regarded as a cytoprotective adaptive response to radio- or chemotherapy, particularly in cancer stem cells [86–88]. Basically, chloroquine and related drugs lead to accumulation of non-functional autophagic vacuoles, thus inhibiting autophagy at its late stages [89, 90]. Indeed, chloroquine has been demonstrated effective in inhibiting cancer stem cell growth in triple negative breast cancer [91] as well as in other neoplastic pathologies [92, 93]. On this basis, antimalarial drugs have been shown to be effective in inhibiting glioma and GBM cell growth in vitro and in vivo in combination with TMZ [94–96], and several clinical trials have been conducted [93, 97–99]. In the context of brain tumors, the derivative quinacrine, employed in the therapy of cerebral malaria, should be also considered for clinical experimentation, due to its elevated permeability through the Blood-Brain Barrier [100]. A number of clinical trials involving the use of chloroquine and related compounds in GBM therapeutic schemes are listed in Table 1.

### Chlorpromazine and other dopamine receptors inhibitors

Chlorpromazine (CPZ) belongs to the class of tricyclic antipsychotic agents. It is a medication used since the ‘50s to cure psychotic disorders. CPZ acts as an antagonist on different postsynaptic and presynaptic receptors, mainly dopamine receptors D2 (DRD2). Recently CPZ has been demonstrated to have at least two further MoAs, which can suggest its use, alone or in combination, in cancer treatment. Indeed, CPZ acts as: a) a potent and specific inhibitor of the mitotic kinesin KSP/Eg5, thus hindering cancer cell proliferation via mitotic arrest and accumulation of defective, monopolar spindles [101] and b) an inhibitor of the AKT/mTOR signal transduction axis in human glioma cells, thus eliciting autophagic cell death [102]. At present, there are no clinical trials involving the use of chlorpromazine in the treatment of GBM or other brain cancers. Of note, dopamine receptor D4 (DRD4) inhibitors, e.g. fananserin, are presently under investigation for their reported ability to selectively induce autophagy in GBM stem cells, with no detectable toxicity in fibroblasts and only minor effects in normal neural stem cells [103]. Such specificity has been interpreted as an ancestral response to neurotransmitters that could be retained by GBM-derived neural stem cells.

## Conclusions

Although the drugs listed above are admittedly limited in number, most of them are apparently able to interfere with critical signal transduction and/or energy metabolism pathway.

The activity of the mTOR complexes 1 and 2 is pivotal for cancer cells, and mTORC1 inhibitors play a key role in restraining cancer cell growth in GBM [104]. Here, a subset of drug classes candidate for repositioning in cancer therapy, e.g. rapamycin plus its derivatives, metformin plus other oral antidiabetic drugs and chlorpromazine, are known to interfere with the AMPK/mTORC1 axis, which is accountable for most of their cancer cell growth inhibitory effects. On the other hand, GBM cells have been demonstrated to withstand mTOR inhibition by activating glutamine metabolism via glutaminase (GLS) and combined mTOR and GLS inhibition results in synergistic tumor growth inhibition in a GBM preclinical setting [105].

Now, a well-known characteristic of most tumors, including GBM, is metabolic reprogramming [87, 106], historically epitomized by elevated glucose consumption, even in the presence of suitable, physiological oxygen concentrations (Warburg effect) [107, 108]. Indeed, GBMs display disproportionately high glycolytic rates, and this metabolic pathway plays a key role in ATP production [109–111]. Of note, increased energy metabolism, a highly recognized hallmark of cancer [112], appears as a common vulnerable target in a disease, GBM, that by definition manifests itself in so many different forms.

A straightforward link between energy metabolism and the autophagy suppressor mTORC1, is hexokinase II (HK-II), the rate-limiting enzyme that catalyzes the first step of glycolysis. In response to glucose deprivation, HK-II binds physically to mTORC1, thus decreasing its activity and positively regulating protective autophagy. [113]. In cancer cells, the cellular energy sensor AMPK, which plays a pivotal role in this process, is considered a key target for inhibiting the autophagy-based cancer cell survival, also in GBM and other gliomas [59, 114, 115].

The medications described above, far from being target-specific drugs, can rather be considered as a means of “less targeted” therapeutic strategy designed to hit fundamental cancer cell dependencies. Considering the well-known heterogeneity of GBM, such an approach holds great potential, since administration of the aforementioned drugs, in association with the current therapeutic options, could definitely hamper diverse, ground-level cancer cell survival mechanisms, thus providing an incisive strategy for GBM treatment that hinders the selection and subsequent enrichment of resistant cell clones.

We must emphasize that all these medications are essentially well tolerated and, when used for other pathologies, have not been historically involved in hazardous side effects in patients. Possible reasons behind this could be the high sensitivity of cancer cells toward these drugs, based on differential metabolic needs of the cancer phenotype (see above), but also the fact that, in other pathologies the dosage for such drugs could be potentially lower than those required to significantly affect cancer cells [65].

Most of the data here described for these drugs clearly highlight the need for exhaustive basic and translational research to elucidate often poorly understood biological mechanisms underlying the effect of a medication. This is a mandatory step in order to allow the translation to clinical application. Current knowledge demonstrates that often it took decades or also centuries to unravel the full complexity of the interplay between a drug and biological systems. At this point, basic and preclinical research can generate proof-of-concept results to stimulate and promote the generation of robust proof-of-concept clinical trials.

### Advantages of drug repurposing

It is important to note that drugs still under patent, but shelved after unsuccessful or only partially successful clinical trials, are amenable to repurposing. Resources have already been spent to develop these drugs and their resuscitation for similar or novel indications can lead to cost-savings and avoidance of risks related to drug development. Indeed, the knowledge of their well-investigated pharmacokinetic and pharmacodynamic characteristics can spare most of the studies directed to assess drug dosage, safety and side effects.

In addition to the above noted advantages of repurposed drugs to diminish expenses and development time to effectively reach the bedside, repurposing should be considered an invaluable opportunity to treat patients when there is no approved therapy or when a patient has exhausted all available treatment options.

### Obstacles to drug repurposing

Another common characteristic shared by most of these medications is that all of them are inexpensive drugs for which patents have not been submitted, have expired or are about to expire, thus making them lacking of monetary incentives for their off-target development. This is paradoxically an obstacle, making it difficult to obtain funding for investigative research, unless the original molecule is not modified; but this can no longer be referred to as repurposing. There are also the so-called “financial orphan drugs”, for which evidence of safety and efficacy has been envisaged, but no clinical trials can be easily conducted, again mostly due to the absence of financial support. As an example, our group is working on an interesting but not patented small molecule, the kinase inhibitor SI113. This molecule, from preclinical in vivo results, holds great promises in cancer combined treatment, also in GBM [116, 117], but lacks of the financial support needed for clinical experimentation.

Finally, basic and translational research for drug repurposing or orphan drugs should be funded ideally by charitable or government agencies. Valid opportunities in this field are offered by the NIH NCATS program (https://ncats.nih.gov/) or the UK Medical Research Council (MRC; https://www.mrc.ac.uk/). Other countries are also promoting basic, translational and clinical research on drug repurposing.

We strongly encourage intelligent and motivated laboratory and clinical investigations aimed at repurposing old drugs. In this context, funding agencies should be encouraged to support this research, considering the spiraling costs of novel anticancer medications and the consequent non-availability for a great number of patients. Research on repurposed drugs can thus represents a less expensive, safer and often immediately available approach to GBM treatment, as well as in other cancer pathologies, also when further lines of treatment are unavailable or unsatisfactory.
